# Current Prospects for Treatment of Solid Tumors via Photodynamic, Photothermal, or Ionizing Radiation Therapies Combined with Immune Checkpoint Inhibition (A Review)

**DOI:** 10.3390/ph14050447

**Published:** 2021-05-10

**Authors:** Sanjay Anand, Timothy A. Chan, Tayyaba Hasan, Edward V. Maytin

**Affiliations:** 1 Department of Biomedical Engineering, Lerner Research Institute, Cleveland Clinic, Cleveland, OH 44195, USA; 2 Dermatology and Plastic Surgery Institute, Cleveland Clinic, Cleveland, OH 44195, USA; 3 Cleveland Clinic Lerner College of Medicine, Case Western Reserve University, Cleveland, OH 44106, USA; chant2@ccf.org; 4 Center for Immunotherapy and Precision Immuno-Oncology, Cleveland Clinic, Cleveland, OH 44195, USA; 5 Department of Radiation Oncology, Cleveland Clinic, Cleveland, OH 44195, USA; 6 Wellman Center for Photomedicine, Massachusetts General Hospital and Harvard Medical School, Boston, MA 02115, USA; thasan@mgh.harvard.edu; 7 Division of Health Sciences and Technology, Harvard University and Massachusetts Institute of Technology (Harvard–MIT), Cambridge, MA 02139, USA

**Keywords:** photodynamic therapy, photothermal therapy, radiation therapy, immunotherapy, immune checkpoint inhibition, murine models, clinical trials

## Abstract

Photodynamic therapy (PDT) causes selective damage to tumor cells and vasculature and also triggers an anti-tumor immune response. The latter fact has prompted the exploration of PDT as an immune-stimulatory adjuvant. PDT is not the only cancer treatment that relies on electromagnetic energy to destroy cancer tissue. Ionizing radiation therapy (RT) and photothermal therapy (PTT) are two other treatment modalities that employ photons (with wavelengths either shorter or longer than PDT, respectively) and also cause tissue damage and immunomodulation. Research on the three modalities has occurred in different “silos”, with minimal interaction between the three topics. This is happening at a time when immune checkpoint inhibition (ICI), another focus of intense research and clinical development, has opened exciting possibilities for combining PDT, PTT, or RT with ICI to achieve improved therapeutic benefits. In this review, we surveyed the literature for studies that describe changes in anti-tumor immunity following the administration of PDT, PTT, and RT, including efforts to combine each modality with ICI. This information, collected all in one place, may make it easier to recognize similarities and differences and help to identify new mechanistic hypotheses toward the goal of achieving optimized combinations and tumor cures.

## 1. Introduction

Cancer, one of the most serious public health problems, has been precisely described as “The Emperor of All Maladies” [1]. The incidence of cancer is increasing worldwide at an alarming rate, with approximately 1.9 million cases diagnosed and 608,570 cases of death expected in the United States alone, according to American Cancer Society estimates for 2021 [2]. Numerous modalities for cancer treatment are currently in use, including chemotherapy, hormonal therapy, and immunotherapy. Several treatments that employ various wavelengths of radiation, from short wavelengths (radiation therapy, RT), visible wavelengths (photodynamic therapy, PDT), or infrared/heat (photothermal therapy, PTT), are also available and undergoing rapid research and development in an attempt to better manage cancer progression and mortality. Despite best efforts, metastatic spread is often undetected until the disease is very advanced, resulting in cancer treatment failure and accounting for nearly 90% of cancer-related mortality. When treatment fails, each of the individual treatment modalities mentioned above can be used for palliation in patients with advanced metastases. However, the extension of survival is often modest, pointing to a need for additional approaches in order to cure cancer. In principle, we need therapeutic strategies that offer high tumor-specificity, minimize off-target normal tissue damage, and achieve long-term cure. Toward the latter goal, research over the past few decades has led to new immunotherapeutic approaches that have been creating much excitement because they exploit the body’s natural defense systems in order to target tumor cells [3,4,5]. Some immunotherapy approaches under investigation include vaccine therapy, cytokine therapy, and most recently, immune checkpoint blockade (ICB) therapy, also known as immune checkpoint inhibition (ICI), which targets cell membrane receptors (such as programmed cell death protein 1, PD-1, programmed cell death protein 1 ligand 1, PD-L1, and cytotoxic T lymphocyte antigen 4, CTLA4) expressed on the surface of tumor cells and tumor-infiltrating immune cells, and whose interactions regulate anti-tumor immune responses [6,7,8,9,10]. While ICI is able to bring about complete cures in some cancer patients, the actual proportion of patients who respond to ICI is unfortunately rather small. This has led to efforts to further stimulate therapeutic responses by combining ICI with more traditional therapies such as chemotherapy, or with radiation-based modalities such as the three mentioned above (PDT, PTT, and RT) [11,12,13,14,15,16]. Research combining ICI with the radiation-based strategies (light, heat, or ionizing radiation) is currently at a very early stage, and the findings are being published in widely disparate specialty journals. However, there could be great value in considering these modalities side by side, i.e., comparing the ability of each treatment to stimulate anti-tumor immunity, and asking whether those changes are leveraged by ICI administered at the appropriate time, resulting in improved therapeutic outcomes. A recent study by our group, in addition to a few studies by others, demonstrated that anti-tumor immunity generated by PDT may play a relatively larger role in the therapeutic outcomes, as compared to direct PDT-induced cell death within the primary tumor, than was previously thought [17,18,19,20,21]. This has major implications because the development of long-term anti-tumor immunity is the desired outcome and ultimate goal for generating durable cancer cures. In this review, we have collected the existing literature pertinent to PDT, PTT, and RT, and described what is known about how each treatment contributes to the development of anti-tumor immunity. We have also described preclinical and clinical studies in which PDT, PTT, or RT were combined with ICI, and the outcomes of those studies. ICI combination with currently available cancer treatment options is a rapidly evolving area. While our review is by no means exhaustive, we hope that by providing information about ICI and the three different radiation-based modalities all in one place, that commonalities and differences may become apparent, possibly leading to insights about how each tissue-damaging approach might best be combined with ICI in order to improve cancer treatment outcomes.

## 2. Immune Checkpoint Inhibition Therapy

Tumors that are resistant to mainline or monotherapies such as chemotherapy and RT often carry a treatment challenge by the upregulation of inhibitory genes and pathways which favor tumor growth in an immunosuppressive tumor microenvironment. Another challenge for the success of immunotherapy is the uncertain relationship between the tumor and its host immune microenvironment, a hot area in the contemporary cancer immunotherapy research field. The majority of tumors thrive in their host environment by neutralizing anti-tumor immunoregulatory signals, such as PD1/PDL1 and CTLA4, that block the cytotoxicity of immune cells and result in immunotherapy failure. In the past two decades, a therapeutic approach called immune checkpoint inhibition (ICI), also known as immune checkpoint blockade (ICB), has been developed to reduce or overcome these inhibitory factors and has been successfully translated to the clinic in combination with mainline treatment therapies for various cancers [22,23,24,25,26]. Immune checkpoint molecules are a subset of inhibitory receptors on the surface of both tumors and T cells which antagonize T cell-mediated killing, thereby evading immune recognition and favoring tumor growth. Some of the most common immune checkpoint receptors are PD1, PDL1, CTLA4, indoleamine-pyrrole 2,3-dioxygenase (IDO) and lymphocyte-activation gene 3 (LAG3) [9,27,28]. PD1, also known as PDCD1 and CD279, is a transmembrane receptor expressed mainly on activated T cells and B cells, and in some cases on macrophages, natural killer cells, and cells of myeloid lineage. PD1 is expressed during T cell activation to counter positive signals that occur through T cell receptor (TCR) and CD28. PD1 engages a specific set of ligands, either PD-L1 (also known as CD274 and B7-DC) or PD-L2 (also known as CD273 and B7-DC), which are expressed on a variety of cell types including cancer cells [8,29,30]. Ligand-bound PD1 receptors function as “brakes” or “immune checkpoints” for T cell-mediated adaptive immune responses, a signal that effector T cells must overcome to exert their cytotoxic activities [30]. CTLA4, also known as CD152, is another negative regulator (and the first one identified) which is induced in Tregs and also in some other T cell types. CTLA4 directly competes with the T cell co-stimulatory activator CD28 for the ligands CD80 (B7-1) and CD86 (B7-2) [8,31]. Blockade of PD1 or CTLA4 by immune checkpoint inhibitory agents (anti-PD1 or anti-CTLA4 antibodies) has been shown to restore tumoricidal activities of T lymphocytes and enhance the therapeutic effects of mainline monotherapies (e.g., RT or chemotherapy) when given as a combination therapy [9,25]. This new approach has revolutionized cancer therapy over the past decade. To date, immune checkpoint inhibitors targeting PD1/PDL1 and CTLA4 have transformed the care of patients with advanced-stage cancers, most effectively for melanoma, renal, head and neck, bladder, and Hodgkin lymphoma [9,27,28].

## 3. Photodynamic Therapy (PDT)

Photodynamic therapy (PDT) is a treatment modality that has been successfully utilized to treat cancer and non-cancerous conditions in the clinic [20,32,33,34]. PDT is a multi-step procedure that involves systemic or topical administration of a light-sensitive photosensitizer (PS), its selective uptake by the tumor, followed by excitation of the PS within the tumor tissue by illumination with visible light. The light source can be either a broadband source or a laser of the appropriate wavelength, i.e., corresponding to a major peak within the absorption spectrum of the PS. The energy generated from the excited state of PS in the presence of oxygen results in the production of cytotoxic singlet oxygen (^1^O_2_) and reactive oxygen species (ROS), triggering a cascade of events leading to tumor cell death and the destruction of tumor vasculature [32,33,35,36,37,38]. The therapeutic effects of PDT depend upon the cumulative response of three well-characterized and mechanistically linked events that occur in sequence. First, PDT directly kills cancer cells by triggering signaling cascades that lead to cell death via apoptosis, necroptosis, autophagy and/or pyroptosis [32,36,38]. Then, PDT-mediated damage of tumor-associated vasculature limits the blood supply and induces hypoxia, resulting in tumor destruction by starvation [39,40,41,42]. These two events are directly responsible for destruction of the primary tumor, activation/release of damage-associated molecular patterns (DAMPs), and the production of cellular debris which primes a third event involving the immune system. This third event, the triggering of inflammation and activation of the immune system, can last from days to weeks and can eventually exert a systemic (abscopal) effect; the latter constitutes the physiological basis for the concept of PDT-generated vaccines for cancer therapy [12,43,44,45,46,47].

### 3.1. PDT-Induced Immunogenic Cell Death (ICD) and Activation of Damage-Associated Molecular Patterns (DAMPs)

Over the past decade, it has been realized that certain chemotherapeutic drugs and cancer therapies, such as PDT, PTT, and RT, induce a form of cell death which triggers an immune response, which is hence referred to as immunogenic cell death (ICD) [36,47,48,49,50]. ICD activates innate and adaptive arms of the immune system, resulting in efficient elimination of tumors by generating long-term immunological memory [51,52]. The immune response generated by ICD is determined by the antigenicity and adjuvanticity of target cancer cells. Antigenicity of the tumor cells is determined by the tumor-specific antigens (TSA), which are essentially PDT-generated tumor debris. The ICD is usually accompanied by the release of adjuvant-like DAMPs which reside in the cells as a part of their normal functions, but once released, act as danger signals [53,54]. DAMPs, either secreted or exposed extracellularly on the surface of dying cells, are recognized by innate pattern recognition receptors (PRRs) such as Toll-like receptors (TLRs) expressed on immune cells, thereby promoting the recruitment of antigen-presenting cells (APCs) [50,52]. Dendritic cells (DCs), being the predominant APCs in most scenarios, take up and process the TSAs and present them to naïve T cells, thereby activating long-term adaptive immunity [36]. The list of DAMPs is continuously growing and includes calreticulin (CRT), high-mobility group box 1 (HMGB1), heat shock proteins (HSPs) 70 and 90, and ATP as some of the common members activated following PDT [21,53,54].

### 3.2. PDT-Induced Inflammation and Activation of Innate Immunity

PDT-induced oxidative stress and cell death trigger an acute inflammatory response that is often seen as edema at the treatment site. Localized inflammatory responses following PDT involve the upregulation and release of inflammatory cytokines such as interleukin 1 beta (IL-1β), IL-6, tumor necrosis factor alpha (TNFα), macrophage inflammatory protein 2 (MIP2) or chemokine (C-X-C motif) ligand 2 (CXCL2), and activation of complement protein C3 [20,36,47,55]. A rather complex balance between the levels of pro-inflammatory and anti-inflammatory cytokines has been linked to the anti-tumoral immune response following PDT. For example, blockade of pro-inflammatory cytokine IL-1β levels diminished the therapeutic effects of PDT [56], but neutralization of transforming growth factor beta (TGFβ) or IL-10 by antibody depletion significantly enhanced the therapeutic effects of PDT [40]. Release of inflammatory cytokines results in the rapid infiltration of immune cells at the site of damage; these cells attack and remove dying tumor cells. Neutrophils are the first population of cells of the innate immune system to enter the damage site, followed by macrophages, DCs, natural killer (NK) cells and lymphocytes. This resets a “cold” tumor microenvironment (non-immunogenic, immunosuppressive) into a “hot” (immunogenic) tumor microenvironment [12,47,57,58,59]. 

Neutrophils, being the most predominant leukocytes early on, have been reported to accumulate in high numbers within less than 5 min following PDT, and remain present at the site until 24 h post-PDT [21,57]. In addition to infiltrating treated tumors, neutrophils have also been reported to accumulate in tumor-draining lymph nodes (DLN). The induction of IL-17 levels by T helper cells (Th17) following PDT has been linked to this migration of neutrophils [60,61]. By secreting alarmins and TNFα, neutrophils have also been reported to help with the maturation and activation of DCs, which trigger adaptive immunity by the stimulating of CD8+ cells [60,62]. 

Dendritic cells (DCs) are the major APCs in the PDT-treated tumor microenvironment (TME), where they phagocytize and process the tumor cell debris, differentiate into APCs, and present TSAs to naïve T cells, resulting in the activation and proliferation of T cells involved in long-term adaptive immunity. Accumulation of CD11c+ and CD1a+ DCs in the treated tumor sites have been reported around 24 h post Photofrin-PDT and Aminolevulinic acid-mediated PDT (ALA-PDT), respectively [63,64]. A recent study by Lamberti et al. showed the critical involvement of the interferon 1 (IFN-1) pathway in regulation of the functions of DCs. PDT-treated B16-OVA murine melanoma cells induced IFN-1-dependent maturation of DCs by enhancing co-stimulatory signals (CD80 and MHC-II) and tumor-directed chemotaxis [65]. 

Another class of immune cell in the TME is macrophages, also referred to as tumor-associated macrophages (TAMs), which differentiate from monocytes and acquire an ability to activate immune effector functions following PDT [21,66,67,68]. In an unperturbed tumor microenvironment, the majority of macrophages belong to an anti-inflammatory M2 phenotype that promotes immunosuppression, growth, angiogenesis and metastasis. Following PDT, the majority of M2 macrophages are removed from the TME and replaced by a fresh population of M1 macrophages, derived from monocytes present in the tumor and surrounding vasculature. These M1 macrophages provide an immunostimulatory environment by secreting pro-inflammatory cytokines (IL-1, IL-6, IL-12 and TNFα) that promote tumor regression [68,69,70,71]. 

Natural killer (NK) cells are another type of cytotoxic lymphocyte, part of the innate immunity system that responds to local inflammation following PDT. Studies by Belicha-Villanueva et al. using human and murine colon carcinoma cells showed an increased expression of MHC class I-like molecules (MICA) and natural killer group 2D (NKG2D) ligands following PDT, which corresponded to enhanced NK cell-mediated killing [72]. These two molecules serve as ligands for activation receptors on NK cells that support their roles in anti-tumor immunity [47]. Additionally, Kabingu et al. reported that the reduction in distant tumors by CD8+ T cells, following PDT of a primary tumor (abscopal effect), was improved in the presence of NK cells, supporting the role of these cells in PDT-mediated anti-tumor immunity [18]. 

### 3.3. Activation of the Adaptive Immune System by PDT

PDT-induced differentiation, activation, and transformation of DCs into APCs is the step which connects the innate and adaptive arms of the immune system; adaptive long-term immunity involves CD4+ (helper), CD8+ (cytotoxic) and regulatory (Tregs) T lymphocytes [12,20,47]. The observation that anti-tumor effects exerted by ICD-inducing therapies involve DC recruitment and activation has resulted in the combination of DC-based immunotherapies with PDT to enhance the treatment outcome. A study by Ji et al., using a murine model of cutaneous SCC, showed the enhancement of anti-tumor activity of DC-based vaccines in mice by immunogenic apoptotic cells induced by ALA-PDT. Higher levels of IFNγ and IL-12 and the suppression of immunosuppressive IL-10 were reported to be associated with functional DC maturation and T cell proliferation [73,74]. A role for adaptive immunity in generating anti-tumor responses after PDT was established by observing a diminished or absent long-term tumor treatment response in immunocompromised mice, and an increase in PDT efficacy after replenishment of these mice with bone marrow or T cells from immunocompetent mice [67,75]. PDT can activate both B cell- (humoral) and T cell-mediated anti-tumor immune responses; however, while both types of responses have been investigated, the major research focus over the past three decades has been upon immune responses involving T lymphocytes [75,76,77]. For T cells, when mature dendritic cells are activated following PDT, the APCs interact with T cells through a complex mechanism of co-stimulation that involves major histocompatibility antigens (MHC class I or II) on the APC, and the T cell receptor (TCR) and co-receptors CD8 or CD4 on the T cell; this process ensures that the proper type of antigen is presented to the appropriate class of T cell, thereby avoiding the development of autoimmunity. Regarding MHC, there are two separate antigen-loading pathways: (1) MHC-I displays peptides that are endogenously derived (e.g., capsid proteins in virally infected cells) within almost any kind of cell, including cancer cells; (2) MHC-II displays peptides that are produced within lysosomes of immune system cells after they ingest foreign proteins (“exogenous” peptides). MHC-I antigen complexes bind to the TCR only when also bound to CD8, thereby activating CD8+ T cells that play a major role in anti-tumor immunity by their tumoricidal/cytotoxic properties. MHC-II complexes will bind TCR only on T cells that express CD4; activation of these CD4+ T helper T cells plays a supportive role in amplifying the adaptive immune response [77,78,79,80,81]. 

Interactions between APCs and naïve T cells can lead to the development of several different subclasses of T cell subsets. Anti-tumor responses after PDT are generally thought to involve three subsets of T cells, namely: (1) CD8+ cytotoxic T lymphocytes (CTL); (2) CD4+ T helper (Th) cells; and (3) regulatory T cells (Tregs). A role for CD8+ CTLs in PDT-induced anti-tumor immunity was first demonstrated by Korbelik et al., who showed that the depletion of CD8+ T cells in EMT6 mammary carcinoma model resulted in a 50% reduction in tumor clearance compared to controls [17]. Similarly, the adoptive transfer of CD8+ T cells from PDT-cured animals protected naïve recipients from cancer cells of the same origin [82]. PDT-induced elevations in the number of CD8+ T cells and increases in their antigen-specific cytotoxic activities have been reported in several preclinical studies; for example, Abdel-Hady et al. reported a direct correlation between treatment response and increased levels of CD8+ cells in lesions following PDT [19,21,60,83,84]. 

The second class of T cell involved in PDT anti-tumor responses, the CD4+ T cells, facilitate the activation of B cells and CD8+ T cells. Involvement of three subtypes of T helper cell populations, i.e., Th1, Th2 and Th17, have been reported in PDT-induced anti-tumor immunity [85,86]. Th1 cells, characterized by the production of IFNγ, can activate CTLs and mediate direct cell killing by the release of cytokines and activation of death receptors on tumor cells [87,88]. Th2 cells secrete cytokines such as IL-4, IL-5, IL-9, and IL-13, which regulate humoral immunity and coordinate immune responses to extracellular pathogens by B cell isotype switching [89]. Th17 cells, defined by their secretion of IL-17 cytokine, are interesting due a dichotomy related to their origin. In an inflammatory TME, levels of TGFβ regulate the differentiation of T cells into either Tregs or Th17 cells. While low levels of TGFβ promote differentiation into Th17 cells, high levels favor their differentiation into Tregs [90]. Experimental immune-depletion of CD4+ cells in mice has shown mixed results. When Korbelik et al. used antibodies against CD4, CD25 and a combination of both to deplete T helper cells, a reduction in treatment response was seen [17,67]. However, a study by Kabingu et al. showed no effect of CD4+ T cell depletion on therapeutic response and systemic anti-tumor immunity [18]. 

The third subtype of T cells involved in anti-tumor responses after PDT are a unique subpopulation in the CD4+ category which are regulatory or suppressive in nature; they are also referred to as suppressor T cells, or Tregs. The most common type of Tregs is CD25+ CD4+ FoxP3+ T cells [91]. At a molecular level, these cells constitutively express high levels of the transmembrane protein CD25, CTLA4, and forkhead box P3 (FoxP3) transcription factor, also known as scurfin [92,93]. By suppressing the differentiation of effector T cells (Teff), Tregs maintain the Teffs in an intermediate stage by favoring IL-2 production. Tregs, by withholding IL-2 and producing TGFβ, prevent full T-effector differentiation during the acute phase of the CD8+ T cell response, blocking differentiation into tumor-specific cytotoxic CD8+ T cells [77,94]. Tregs were shown to be involved in anti-tumor immunity induced by PDT, in two studies showing that suppression of Tregs using a cyclophosphamide-PDT combination led to improved therapeutic efficacy (enhanced tumor regression and long-term survival) in murine models of reticulum cell carcinoma and colon carcinoma [95,96]. Oh et al. showed that intra-tumoral depletion of Tregs using CD25-targeted photodynamic therapy in a mouse melanoma model induced antitumor immune responses, possibly due to increased infiltration of CD8+ effector T cells and the expression of interferon gamma (IFNγ) and CD107a, a marker of cytotoxicity [97].

**Figure 1:**
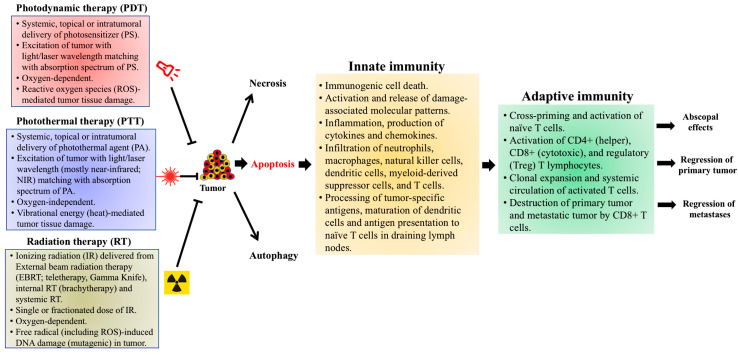
Photodynamic therapy- (PDT), photothermal therapy- (PTT), and radiation therapy (RT)-induced effects on anti-tumor immunity, based on preclinical studies in murine tumor models. Treatment of tumors with PDT, PTT or RT leads to cell death within the primary tumor by apoptotic, necrotic and autophagic mechanisms. Apoptosis induced by these therapies also generates an immune response, referred to as immunogenic cell death (ICD), within the tumor microenvironment (TME). Damage-associated molecular patterns (DAMPs), expressed on the surface of dying cells and released into the TME, promote the recruitment and maturation of antigen-presenting cells (APCs), primarily dendritic cells (DCs). Various cytokines and chemokines (IL-1β, IL-6 and TNFα), released by the photodamaged cells, induce local inflammation and recruit cells associated with innate immunity (neutrophils, macrophages, natural killer cells, and mast cells). The DCs engulf and process tumor-specific antigens (TSAs), then migrate to draining lymph nodes (DLNs) to present the processed TSAs to naïve T cells, thereby triggering the adaptive arm of anti-tumor immunity. Activated T cell subsets (CD4+, CD8+ and FoxP3+) undergo clonal expansion and differentiation within the TME, mediating tumor regression via cytotoxic activities of CD8+ cytotoxic T lymphocytes (CTLs). Optimally, these activated T cells may enter the systemic circulation and travel to distant metastases, mediating a more widespread (abscopal) effect. The immunological events discussed here were observed in PDT-treated murine tumor models, but similar mechanisms of anti-tumor immunity have also been reported in pre-clinical studies using PTT and RT (see text for details).

A brief summary of the important properties of PDT, PTT and RT, and of subsequent events that result in induction of anti-tumor immunity (based on studies in murine tumor models) is provided in Figure 1.

### 3.4. Combination of Immune Checkpoint Inhibition with PDT

**Figure 2:**
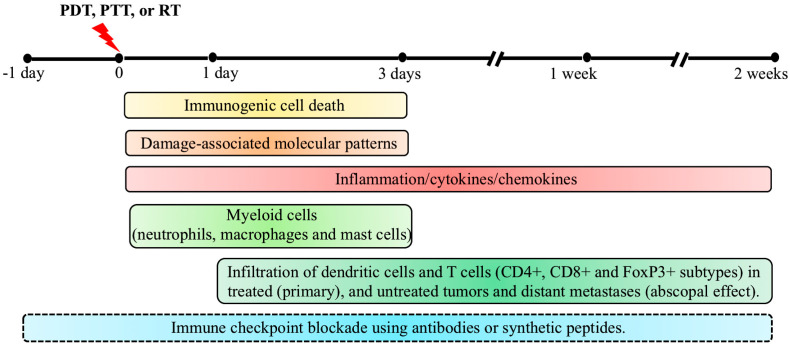
Timeline of immunological events contributing to anti-tumor immunity by photodynamic therapy (PDT), based on preclinical studies in murine tumor models. Following PDT, tumor cells undergo cell death mainly by apoptosis, necrosis, and autophagy as PDT’s primary therapeutic mechanism. In parallel, immunogenic cell death (ICD) triggers anti-tumor immunity by inducing inflammation and activation and release of damage-associated molecular patterns (DAMPs). While effects of ICD and DAMPs can last from 1 day to 3 days post-PDT, involvement of cytokines and chemokines can last from the time of light exposure until 2 weeks post-treatment in some cases. A robust neutrophil infiltration occurs within minutes following PDT, followed by infiltration of macrophages and mast cells, most prominently in the first 3 days post-PDT. One day post-PDT, dendritic cells (DCs) along with lymphocytes start infiltrating the treated tumor site. Maturation of DCs by exposure to tumor-specific antigens, presentation of processed antigens to naïve T cells, and elevated levels of TNFα, IFNγ and IL-6 in the tumor microenvironment (TME) trigger the adaptive immune response during the two weeks post-PDT. Activated T cells (CD4+, CD8+ and FoxP3+) undergo clonal expansion and reach the primary tumor and metastatic sites through the systemic circulation to induce the regression of primary and metastatic tumors (abscopal effect). The combination of immune checkpoint inhibition (ICI) with PDT, PTT or RT has been explored in pre-clinical models by injecting antibodies against PD1/PDL1, CTLA4 and IDO, at a variety of times (from 1 day prior up to 2 weeks post-therapy) with the reinjection of antibodies every 2–3 days until the endpoint. The optimal sequence and timing of these combinations is still under exploration. Although the timeline and immunological events discussed above were observed in PDT-treated mouse models, similar mechanisms of anti-tumor immunity have also been reported in pre-clinical studies using PTT and RT, as described in the text.

Tumors that are resistant to PDT as a monotherapy pose a therapeutic challenge by the upregulation of inhibitory genes and pathways which favor tumor growth in an immunosuppressive tumor microenvironment. Combinations of ICI with PDT have been explored mainly in preclinical studies in the past decade for their potential to overcome the inhibitory effects of immune checkpoints, with the ultimate goal of future translation into the clinic. In this section, we describe a few preclinical studies investigating the combination of ICI with PDT to improve the therapeutic efficacy. A combination of PDT and ICI using antibodies against PD1/PDL1, CTLA4 and IDO have been investigated in preclinical studies using murine cancer models of breast, colon, renal, lung and skin, to show significant improvements in therapeutic efficacies [98,99,100,101,102]. For example, Zhang et al. used chlorin 6-mediated PDT combined with either an inhibitor for PD1/PDL1 interaction called Bristol Mayers Squibb 202 (BMS-202), or an anti-PDL1 antibody treated 4T1 murine breast cancer model and showed that tumor regression was associated with the inhibition of lung metastasis. The therapeutic effects by combination regimens were possibly achieved by enhanced maturation of DCs and infiltration of CD8+ T cells, along with increased levels of IFNγ, IL-6, and TNFα cytokines [103]. In another study using a mouse model for renal carcinoma which developed lung metastasis, O’Shaughnessy et al. showed that the combination approach had synergistic effect over tumor regression and metastasis to lungs, compared to the outcome with either treatments given alone. Furthermore, CD8+:Tregs and CD4+:Tregs ratios were increased in both primary tumors and lung metastasis in the combination treatment mice [99]. Details of a few recent studies using immune checkpoint inhibition combination with PDT in murine tumor models showing the elimination of primary and distant (abscopal effect) tumors, reduction in metastases, and the involvement of immune cells in the observed outcome have been listed in Table 1. A timeline of immunological events that contribute to anti-tumor immunity after PDT, along with the time frame for effective ICI, is shown in Figure 2. 

Finally, when thinking about how to optimize PDT and ICI for cancer treatment, there is some literature suggesting a role for vascular endothelial growth factor (VEGF). One of the most important clinical advances in the last few years has been the combination of anti-PD1 agents (e.g., pembrolizumab) with VEGF-targeting tyrosine kinase inhibitors such as axitinib [110,111,112] and lenvatinib [113]. In animal tumor models, PDT has been shown to transiently increase VEGF expression [114,115], providing a potential rationale for why anti-VEGF blockades could be helpful when designing effective combination therapies. However, any new approach must be approached with caution due to competing effects of PDF, VEGF, and the immune system. For example, because tumor-derived VEGF encourages the growth of lymphatic vessels, and the latter potentially increases the risk of metastasis, one might think that PDT-induced damage of tumor-draining lymphatics would be helpful. However, a recent study in a murine breast cancer model showed that verteporfin PDT does indeed destroy lymphatic vessels—a combination treatment with anti-VEGF blockade or with lenalidomide (a lymphangiogenesis inhibitor) actually reduced tumor responsiveness to PDT and abrogated the potentiation of therapy by anti-PD1 monoclonal antibodies (mAbs); these effects were largely due to the reduced migration of DCs from the tumor to the DLNs [105]. 

Clinical studies that use a combination of ICI with PDT are very few at the current time, consisting of one published case report and one clinical trial in ClinicalTrials.gov (accessed on 24 April 2021), as described in more detail in Section 5.4).

## 4. Photothermal Therapy (PTT)

Photothermal therapy (PTT), a non-invasive, local treatment modality for cancer, utilizes a combination of light-absorbing photothermal agents (PTAs) and their wavelength-matched light or laser source to generate heat, which results in the thermal ablation of tumors, causing cell death [116,117]. Similar to photosensitizers (PS), during PDT, PTAs absorb energy from incoming photons and undergo a transformation from an electronic ground state to an excited state. Upon returning to its ground state by vibrational relaxation, the excited photothermal agent emits kinetic energy, heating the surrounding tissue, and causing thermal damage to the tumor microenvironment. Unlike PDT, in which PS is excited with a specific wavelength light to generate ROS in the presence of oxygen, PTT does not require oxygen in order to interact with target cells or tissues [14,116,118]. Recently developed PTAs use longer wavelengths of light, which not only penetrate deeper into the tissue, but are also less energetic and therefore less harmful to surrounding cells and tissues. The list of novel PTAs, comprising engineered nanomaterials with unique activation mechanisms to provide tumor-specific targeting and reduce adverse off-target effects, is continuously evolving [13]. Due to their promise of limited side effects and relatively low drug resistance, PTT agents have evolved through four generations, including precious metal nanoparticles such as Au, Ag, Pt, carbon nanorods and graphene, metal and non-metal compounds such as CuS and ZnS, and organic and inorganic nanomaterials such as Prussian blue, Indoline green, and organic semiconducting pro-nano-stimulants (OSPSs), which are still in an exploratory phase for research and clinical applications [119].

Similar to PDT, nanoparticle-based PTT for the treatment of cancer offers the following unique and advantageous features: (i) combination of a near-infrared (NIR) laser with nanoparticle-based photothermal agents allows target-focused and deeper penetration of the activating signal [119,120]; (ii) tumor-specific target molecules (peptides or nucleic acids) included in the nano-formulation can offer tumor-specific targeting and avoid off-target systemic effects [118,121]; (iii) by combining PTT with interventional technologies for the delivery of light, PTT is not limited to superficial tumors, but can treat internal malignancies such as prostate and pancreatic cancer [13,122,123,124]; (iv) PTT agents can be used for image-guided therapy using theranostic nanoparticles [13,125,126,127]. In preclinical studies, PTT has been successful as a monotherapy or in combination with other therapies for the treatment of several malignancies including breast cancer, melanoma, and liver cancer in murine models [116,118,128]. Based upon such preclinical studies, nanoparticle-based PTT has been successfully translated into the clinic to treat low- or intermediate-risk localized prostate cancer [123,129]. Several clinical trials using AuroLase, a type of PTT that combines silica–gold nanoshells (AuNS) with NIR light to treat tumors of the head and neck, lung and prostate have been reported as described in reference [130]. Similar to PDT, PTT causes destruction of the peritumoral extracellular matrix and vasculature, induces inflammation, and releases tumor antigens which trigger an anti-tumoral immune response by the recruitment of endogenous immune cells. Evidence that PTT elicits ICD and activation of inflammatory response, followed by innate and adaptive immune responses, is discussed in the following sections.

### 4.1. PTT-Induced Immunogenic Cell Death (ICD), Activation of Damage-Associated Molecular Patterns (DAMPs) and Activation of Anti-Tumor Immunity

Depending on the combination of photothermal agent and activating light wavelength utilized, PTT raises the temperature of the tumor microenvironment to 41–48 °C, which causes tissue damage, including damage to cellular architecture, degradation of proteins and nucleic acids (DNA/RNA), and eventually results in apoptosis [14,131,132]. Although both apoptotic and necrotic damage has been reported following PTT, it appears that one or other pathway may be favored, depending upon the hyperthermic temperature achieved during therapy [14,116]. When the temperature of the tumor microenvironment is raised to 41 °C, a heat shock response is initiated, which mitigates the effects of thermal damage on cellular machinery [133]. A rise in temperature to between 42 and 46 °C results in irreversible tissue damage and promotes apoptosis. Sustained hyperthermia (42–46 °C) that lasts beyond 10 min results in tissue damage by necrosis. A temperature above 60 °C often triggers instantaneous cell death by the denaturation of cellular components [116,134].

As discussed in the PDT section, cell death induced by PTT also triggers the induction of ICD that involves the release of TSAs and DAMPs from dying tumor cells, followed by the maturation of DCs that can activate T cells and trigger anti-tumor immunity. However, unlike PDT, PTT may only induce ICD within a specific thermal window. For example, a study by Sweeney et al. using Prussian blue nanoparticles (PBNP) for PTT in neuroblastoma cells showed that the induction of ICD was specific to an optimal thermal dose, at which the release of DAMPs (calreticulin, HMGB1 and ATP) was observed and a potent anti-tumor response was achieved. If the thermal dose was too low or too high, tumors cells were eliminated by cell death after PPBNP-PTT, but the dying cells did not trigger any ICD-mediated anti-tumor response [135]. 

In addition to inducing ICD, PTT can also activate innate immunity through macrophage reprogramming. Using a uniformly conjugated polymer nanoparticle for PTT, Wang et al. showed activation of a pro-inflammatory immune response (M1 macrophages) and inhibition of tumor growth in a murine tumor model [136]. Another study by Yu et al. used magnetic Fe_3_O_4_ photothermal nanoparticles (MNPs) coated with myeloid-derived suppressor cell membranes (MNP@MDSC) for PTT, demonstrating the enhancement of ICD (upregulation of HMGB1 and calreticulin) and reprogramming of infiltrating macrophages that involved increased CD86+ M1 macrophages and decreased CD206+ M2 macrophages in a B16/F10 murine melanoma model [137]. However, some recent studies utilizing conventional and nanomaterial-based PTAs have shown that immune-stimulation induced by PTT alone was not sufficient to effectively activate long-term anti-tumor immunity. Therefore, new efforts to combine nanoparticle-based PTT with immunoadjuvants or other immune response-promoting drugs are underway (see the next section). 

### 4.2. Nanoparticle-Based Photothermal Immunotherapy

The term “photothermal immunotherapy” has been coined to encompass nanomaterial-based PTT that can not only eliminate primary tumors, but also reduce metastasis through sustained anti-tumor immune effects [13,14]. However, in several recent studies, it was shown that immunomodulation by PTT alone was not sufficient to activate long-term anti-tumor response. To overcome this limitation, immune response-promoting drugs have been added as immunoadjuvants to nanoparticle-based photothermal agents [13,14]. Photothermal nanoparticles loaded with immunoadjuvants have been shown to activate both innate and specific immune responses by significantly inducing the infiltration and maturation of NK cells and DCs in the tumor microenvironment, increasing the levels of immune-related cytokines in peripheral blood, and resulting in the inhibition of primary tumor growth and reductions in metastases [13,138,139]. A study by Guo et al., using a hollow copper sulfide nanomaterial for PTT in a murine model of breast cancer, showed no significant activation of the immune system; however, combining their formulation with CpG oligodeoxynucleotides, which activate Toll-like receptor 9 signaling in DCs, significantly increased the infiltration of NK cells and DCs in tumors and DLNs, with an increase in IFNγ and IL-2 secreted by CD8+ T cells in tumors and spleen [140]. A gold nanorod (GNR)-based hybrid nanomaterial (mPEG-GNRs@BSA/R837), involving the functionalization of BSA-bioinspired GNRs with imiquimod (R837, an immunoadjuvant recognized by Toll-like receptor 7), was used by Zhou et al. to treat melanoma in a murine model [141]. PTT with the mPEG-GNRs@BSA/R837 formulation enhanced the levels of cytokines (TNFα, IL-6 and IL-12), mature DCs, and CD8^+^ T cell infiltration. In long-term analyses, prevention of lung metastasis and immunological memory with tumor re-challenge were also reported [141]. Another study by Zhou et al., using single-walled carbon nanotubes (SWCNTs) with an immunoadjuvant glycated chitosan (GC, an immunoadjuvant for the improvement of transport between the epithelium and promotion of phagocytosis) for PTT with a 980 nm laser in a murine model showed regression of the primary tumor, inhibition of metastasis, and a long-term anti-tumor immune response [142]. A detailed review of different types of immunoadjuvants combined with PTT and their therapeutic effects mediated by the immune response has been offered by others [13,14]. 

### 4.3. Combination of Immune Checkpoint Inhibition (ICI) with Photothermal Therapy (PTT)

Immune responses under normal physiological conditions are regulated by checkpoint receptors expressed on the surface of immune cells to maintain immune homeostasis and prevent autoimmunity. Photothermal therapy for cancer as a monotherapy is often insufficient to completely inhibit primary tumor growth, or to prevent distant metastasis; therefore, ICI together with PTT has been explored as a combination immunomodulatory approach for the treatment of tumors refractory to either PTT or ICI alone, in several preclinical studies [13,14,117,143]. A combination of PTT and ICI using antibodies against PD1/PDL1 and CTLA4 has been used in preclinical studies and shows significant improvement in therapeutic efficacies [14,117,144]. For example, Liu et al., using gold nanostar (GNS)-mediated PTT combined with an anti-PDL1 antibody, treated MB49 murine bladder cancer model and showed the complete clearance of primary tumors, along with distant untreated tumors (an abscopal effect) [145]. Treated mice showed long-term immunity (60 days) in re-challenge experiments with MB49 cells [145]. Wang et al. used PTT with SWNTs and demonstrated an increase in CD4+ Tregs with immunosuppressive characteristics [146]; a further combination with anti-CTLA4 antibody could reduce the Tregs and enhance the cytotoxic effects of T cells, thereby reducing the generation of primary tumors and distant metastasis [146]. Similarly, PTT using the organic nanocomposite PLGA-ICG-R837 combined with anti-CTLA4 exerted significant suppressive effects on primary and distant tumors, followed by the generation of memory T cells and inhibition of tumor recurrence and long-term tumor-free survival [147]. Table 2 provides a selected list which includes some other preclinical studies on photothermal immunotherapy that employ photothermal nanomaterials combined with ICI, and immunoadjuvants in some cases, along with study outcomes.

Regarding clinical trials, nanoparticle-based PTT as a monotherapy has been successfully translated into the clinic to treat low- or intermediate-risk localized prostate cancer [123,129] and several clinical trials using AuroLase, a type of PTT that combines AuNS with NIR light to treat tumors of the head and neck, lung, and prostate have been reported [130]. However, regarding combinations of ICI and PTT, no published reports nor any ongoing clinical trials listed on ClinicalTrials.gov (accessed on 24 April 2021) are currently available. 

## 5. Radiation Therapy (RT)

Radiation therapy (RT) using ionizing radiation is a curative treatment for localized cancer and secondary metastasis. On average, 50–60% of patients with early- to mid- stage cancers of breast, prostate, cervical, endometrial, head and neck, lymphoid, etc., receive radiation therapy, either alone or in combination with surgery or chemotherapy [156]. Radiation therapy can be delivered from outside (external-beam radiation therapy; EBRT), by implanting radioactive sources inside the body (brachytherapy), or through systemic administration of radiopharmaceutical agents [157]. At the atomic level, the predominant interaction of tumor tissue with photons released during therapy is the so-called “Compton effect”. After collision of a photon with an orbital electron, both are scattered, and while the photon continues on for additional interactions, the electron begins to ionize due to energy imparted by the photon, thereby allowing chemical reactions and destruction of tissue to occur [158]. 

### 5.1. Radiation Therapy-Induced Cell Death, Immunogenic Cell Death (ICD), and Activation of Anti-Tumor Responses

Ionizing radiation (IR), delivered in the form of X-rays, γ-rays, electrons, or protons, produces ROS and other types of ionizing free radicals upon interaction with tumor tissue, and results in DNA damage. Cells with damaged DNA undergo cell cycle arrest and lead eventually to either cellular senescence or cell death by activating apoptosis, necrosis, and autophagy, depending on the dose and schedule of the radiation therapy [16,159,160,161]. Ionizing radiation triggers cell death by apoptosis via intrinsic and extrinsic apoptotic pathways; the former involves activation of proapoptotic proteins/caspases, whereas the latter involves death receptors followed by downstream caspases, respectively. The DNA damage induced by IR results in cell cycle arrest and induction of senescence, both mediated by the activation of p53, leading to upregulation of p21. Ataxia-telangiectasia mutated (ATM) kinases activated by IR induce autophagy involving active p53 and damage-regulated autophagy modulator (DRAM) and is reviewed in [162]. Similar to PDT and PTT, RT is also known to exert anti-tumor immune responses, resulting in regression of the primary tumor, of distant untreated tumors (abscopal effect), as well as exerting anti-metastatic effects [163,164,165]. The involvement of the host immune system in anti-tumor effects of RT was first demonstrated by Stone et al. in a murine model of fibrosarcoma, showing that the dose of radiation required for tumor control was much higher in immunocompromised mice relative to immuno-sufficient mice [166]. Several studies afterwards elucidated the interplay between RT and anti-tumor immunity, both at the local and systemic levels, the latter being defined as “abscopal effect” [167]. Overall, the cascade of events involving IR-induced ICD are similar to those reported in PDT and PTT sections, and involve the activation of both innate and adaptive immunity. Briefly, the cell death induced by IR results in the release of DNA and RNA into the cytoplasm, triggering the activation of TLRs and transcription of type I interferon (IFN) gene. Type I interferon is essential for the activation of DCs, and for recruitment and regulating the effector function of CD8+ T cells [168,169]. The cytokines and chemokines produced in the tumor microenvironment following IR trigger the infiltration of immune cells such as DCs, macrophages, myeloid-derived suppressor cells (MDSCs), and regulatory and cytotoxic T cells. Activation and release of DAMPs following ICD trigger the activation of APCs, initiating adaptive immune response, reviewed elsewhere [15,16,161,162]. The anti-tumor effects of ICD are determined by the antigenicity and adjuvanticity of target cancer cells. While the antigenicity of tumor cells is determined by TSAs and tumor neoantigens (TNA), the adjuvant-like effects of ICD are mediated by the release of DAMPs [52,170,171]. A recent report by Lhuillier et al., using a 4T1 triple-negative murine breast tumor model, demonstrated radiotherapy-induced upregulation of the expression of genes containing immunogenic mutations in a poorly immunogenic model. Vaccination with neoepitopes encoded by these genes triggered a CD8+ and CD4+ mediated immune response, thereby improving the therapeutic efficacy of RT. The cytotoxic activity was mediated by the upregulation of MHC II molecules and death receptors FAS/CD95 and DR5 on the surface of tumor cells [172]. Dendritic cells, being professional APCs, serve as the link between innate and adaptive immune responses by their ability to stimulate unprimed naïve T cells and perform antigen cross-presentation [74]. A role for irradiated tumor-primed DCs in the prevention of local tumor growth involving CD4+ and CD8+ T cells was shown much before the introduction of the concept of ICD [173,174]. A recent study showed that X-ray-irradiated tumor cell lysates may work as effective antigen/adjuvant sources in DC vaccination studies. DCs, when incubated with X-ray-irradiated tumor cell lysates along with granulocyte-macrophage colony-stimulating factor (GMCF) and lipopolysaccharide (LPS)-containing media, led to reduced infiltration of Tregs, TAMs and MDSCs, along with enrichment of CD3+ T cells and strong infiltration of Th1 cells and CTLs [175]. 

Unlike PDT and PTT, which rely on systemic, local, or topical administration of PS or PTA, respectively, followed by irradiation with light, radiation delivered by an external or implanted source interacts directly with the tumor tissue without being limited by bioavailability, vascular permeability, and retention/efflux issues. Nevertheless, tumor resistance to RT represents an ongoing challenge for radiation oncologists. Possible reasons for this include hypoxia, and the presence of a significant proportion of growth-arrested/slow-dividing tumor cells that escape the therapeutic effects of RT. Normal tissue injury is an inherent consequence of radiation therapy, and hence a key consideration in the treatment design when using ionizing radiation. The effects of radiation on the tumor microenvironment can be regulated by the IR dose and methods of delivery; methods for improving anti-tumor efficacy include accelerated and hyper-fractionation of the radiation dose, in order to improve the tumor-killing effects while avoiding normal tissue damage [157]. In preclinical studies, the use of a high hyper-fractionated dose compared to a high single dose showed an advantage in terms of immunogenic effects of radiation therapy [176]. In addition to immunogenic effects, immunosuppressive effects of IR have been described that can counteract its anti-tumor immune effects. Thus, IR can switch the phenotype of infiltrating macrophages and alter the balance of Tregs and cytokines such as TGFβ, suppressing anti-tumor immunity [177,178,179,180]. In this scenario, radiation therapy alone may not be effective in generating robust immune response. Therefore, a number of combination approaches, including dose fractionation, immunotherapy, and different types of tumor and host factors, are currently being explored in preclinical murine tumor models [157]. One such combination of ICI with radiation therapy has been successfully trialed in preclinical murine models and is currently being utilized in the clinic to improve the therapeutic outcome of radiation therapy for different types of cancers, as described below. 

### 5.2. Combination of Immune Checkpoint Inhibition (ICI) with Radiation Therapy (RT)

In the past decade, anticancer immunotherapy, specifically by ICI, has revolutionized the management of cancer, even in individuals with advanced-stage disease [22,23,24,25,26]. Both ICI and RT involve innate and adaptive immune systems; therefore, the effects of ICI may synergize with those of RT to improve the anti-tumor responses typically observed with either modality alone [15,16,181]. In this section, we discuss both preclinical and clinical studies in which ICI has been used in combination with RT.

### 5.3. Preclinical Scenario

Both the treatment regimens, ICI and RT, often fail to give a significant treatment response, when given individually. Therefore, finding combination treatment strategies to improve the clinical outcome would be highly desirable. There have been several preclinical studies using ICI plus RT in murine models for different types of cancer that have shown promising results. In a conditional Kras-driven genetically engineered mouse model (GEMM) of non-small-cell lung carcinoma (NSCLC), treatment with radiotherapy and an anti-PD1 antibody resulted in significant volume reduction (up to 70%) of the target lesion, and durable tumor regression (up to 12 weeks), along with an increase in inhibitory T cell markers [182]. In another C57BL/6 tumor xenograft mouse model of lung cancer, the combination of anti-PDL1 and IR treatment resulted in tumor regression as compared to either monotherapy alone [183]. In that study, increased infiltration of CD8+ T cells and reduced presence of MDSCs and inducible Tregs were reported only in the combination treatment group [183]. Combination of CTLA4 with RT in a dual murine model of mesothelioma enhanced the tumor regression, relative to either single treatment. Although RT alone increased both Tregs and CTL infiltration in primary tumors, the addition of CTLA4 reversed the proportion of Tregs to effector T cells, with increased CD8+ T cell activation [184]. Many of these preclinical studies used different doses of RT, with different schedules and delivery methodologies. Therefore, a better optimization of the radiotherapy component of the study is still needed for translation into the clinic. The sequence of combination may also be very important, because in a study using the CT26 colorectal murine model, an efficient systemic response to the combination was observed when anti-CTLA antibody was given prior to RT [185]; in contrast, another study using the 4T1 breast tumor model showed efficient tumor regression when anti-CTLA antibody was given after RT [186]. A list of selected preclinical studies using a combination of ICI with RT, and their outcomes, is presented in Table 3.

### 5.4. Clinical Trials with Combination of Immune Checkpoint Inhibition with Radiation, Photodynamic, or Photothermal Therapy

Amongst the three treatment modalities in this review, radiotherapy using ionizing radiation is the only one that has a long track record of widespread clinical use in oncology. Therefore, it is not surprising that physicians and clinical researchers have begun exploring different combinations of RT and various ICI agents, for a variety of cancers. To date, at least two dozen phase I and II trials to evaluate safety have shown that combinations of radiation plus an ICI is generally safe, with the possible exception of increased brain swelling and necrosis in patients with brain metastases treated with combined RT and ICI [16,161]. An excellent current listing of published results from these trials is provided in the supplementary table of the review by McLaughlin et al. [161]. In terms of unpublished results (from ongoing trials that are still recruiting or awaiting analysis), ~20 trials can be found on the ClinicalTrials.gov website (accessed 24 April 2021), as listed in Table 4. These trials involve a variety of internal malignancies, and they feature different brand-name ICI drugs (targeting PD1, PD-L1, or CTLA4, either alone or in combination). They also vary as to whether additional chemotherapeutic drugs are administered, and whether the checkpoint inhibitor is given before or after radiation (Table 4). Especially notable here is the AstraZeneca PACIFIC trial, which used adjuvant durvalumab following chemoradiotherapy for stage III NSCLC. This was phase III data showing that combination chemo/RT and anti-PD-L1 should be the new standard of care [197]. The results of other studies, once available, will provide further insight into the benefits of RT + ICI combination treatments, relative to RT or ICI alone, for the amelioration of human cancer.

Unlike RT and chemotherapy, PDT and PTT are still considered in the broader oncology arena to be palliative or investigational modalities at this time; therefore, the clinical role of combining ICI with PDT or PTT agents has not been widely tested. For PDT, there is an interesting clinical case report in which a patient’s advanced head-and-neck cancer was cured via the administration of PDT (redaporfin/red light) followed by anti-PD1 antibody [198]. However, there are no published studies involving multiple patients. One ongoing PDT clinical trial involving PDT + ICI is listed in ClinicalTrials (identifier NCT04400539); in that trial, lung cancer patients (mesothelioma) will be treated with intrapleural PDT followed by injections of Nivolumab (anti-PD1). For PTT, no current publications nor any listings on ClinicalTrials that describe human trials with PTT + ICI combinations can be found.

## 6. Conclusions

In this review, we have surveyed the available literature on three treatment modalities that employ the electromagnetic spectrum, from very short (RT) to longer wavelengths (PDT and PTT), thereby causing tissue damage and stimulating a number of immune modulatory effects. Ongoing attempts to harness these effects by using immune checkpoint inhibitors were also reviewed. Many similarities and differences between the modalities can be identified. For example, although ICD responses and immune stimulation occur after both PDT and PTT, dose delivery may be a relatively more important factor in PTT, because the response appears to require reaching an optimal temperature range in tissue. Radiation therapy, although generally causing more limited damage targeted to the nucleus (as compared to cellular membrane damage caused with PDT), appears quite capable of inducing strong anti-tumor immune effects.

While our own particular research interest lies in PDT (still an investigational therapy in most human cancers), it is evident that much can be learned by comparison with the other modalities. For example, because RT is widely established and used in clinical practice, considerations of similarities and differences between RT and PDT or PTT could be instructive as ongoing clinical trial results are published and we learn which factors are critical for improving the effects of checkpoint inhibition. Clearly, with such a wide variety of cancers, each with different pathological features and different tumor microenvironments, one can anticipate an important role for each of these photon-involving modalities in combination with ICI in the future.

## Figures and Tables

**Table 1:** A list of selected pre-clinical studies using combinations of immune checkpoint inhibition and photodynamic therapy.

Checkpoint Inhibitor Target	Photosensitizer/Construct	Murine Tumor Model	Immune Effector Cells	Cytokines	Therapeutic/Immune Response	Ref.
PD-1	αvβ6 integrin-specific phthalocyanine dye labeled probe	4T1 breast tumor	DC, CD8+ T cells	IL-1β, IL-12	Reduced primary tumor growth and lung metastasis. Abscopal effect.	[98]
PD-1	Pheophorbide A, given together with a tumor-specific peptide vaccine adjuvanted with TLR5 antagonist	BF16-F10 murine melanoma model	DC, CD8+ T cells	IFNγ	Reduced primary tumor growth and lung metastasis	[101]
PD-L1	IRD700, conjugated to Fab fragment of anti-αCD276 antibody	4T1 breast tumor	CD8+ T cells	Not analyzed	Reduced primary tumor growth and lung metastasis	[104]
PD-L1	EGFR-targeted porphyrin-containing nanoliposomes conjugated with IRDye800CW and DOTA-Gd	Subcutaneous CT26 colon cancer	Not analyzed	Not analyzed	Tumor regression	[100]
PD-L1	Verteporfin	4T1 breast tumor	DC, CD8+ T cells	Not analyzed	Regression of primary tumors by destruction of tumor-associated lymphatic vessels	[105]
PD-L1 and BMS202 PD1/PDL1 inhibitor	Chlorin 6 NPs	4T1 breast tumor	DC, CD8+ T cells	IFNγ, IL-6, TNFα	Regression of primary tumors, reduced lung metastases	[103]
PD1 + PD-L1	WST11	Renal cell carcinoma line that develops lung metastases	CD8+, CD4+FoxP3-T cells	Not analyzed	Regression of primary tumors, reduced lung metastases	[99]
CTLA4	Bremachlorin	Subcutaneous MC38 and CT26 colon cancer double tumor model	CD8+ T cells	Not analyzed	Significant improvement of therapeutic efficacy and survival, abscopal effect	[106]
CTLA4	Nanoparticles simultaneously loaded with chlorin e6 (photosensitizer) and imiquimod (Toll-like receptor-7 agonist)	Subcutaneous CT26 colon cancer	DCs, CD8+, CD4+FoxP3+ T cells	IFNγ, IL-12, TNFα	Therapeutic efficacy with abscopal effect. Prevented tumor recurrence, via immune memory effects	[107]
CTLA4	OR141	Ab1 and Ab12 mesothelioma murine model	CD4+ and CD8+ T cells, DCs	Not analyzed	Inhibition of mesothelioma cell growth	[102]
IDO	Chlorin-based nanoscale metal–organic framework (nMOF)	Subcutaneous B16F10 melanoma and CT26 colon cancer double tumor model.	CD4+ and CD45+ T cells, neutrophils, and B cells	Not analyzed	Local and distant tumor rejection and T cell infiltration of TME. Compensatory roles of neutrophils and B cells in presenting TAAs to T cells	[108]
IDO	Verteporfin	4T1 breast tumor	Myeloid cells	IL-6	Tumor regression	[109]
		E0771 breast tumor				

**Table 2:** A list of selected pre-clinical studies using a combination of immune checkpoint inhibition and photothermal therapy.

Checkpoint Inhibitor Target	Photothermal Agent/Construct	Murine Tumor Model	Immune Effector Cells	Cytokines	Therapeutic/Immune Response	Ref.
PD1	Hollow gold nanoshell (HAuNS)	4T1 breast tumor Colon cancer CT26	CD4+ and CD8+ T cells	IFNγ, IL-2, TNFα	Reduced primary tumor growth and distant metastasis.	[148]
			B cells			
PD1	Black phosphorus quantum dots (BPQDs)	BF16-F10 murine melanoma	DCs, CD4+ and CD8+ T cells	IFNγ, TNFα	Reduced primary tumor growth and inhibition of lung metastasis.	[149]
		4T1 breast tumor				
PD1	A triple-layer nano-system AuNC@mSiO2@ copolymer∩vemurafenib (ASP∩V)	SMM103 melanoma tumors	CD3+, CD4+ and CD8+ T cells	Not analyzed	Primary tumor regression and distant tumor regression by abscopal effect.	[150]
PD1	ZIF-PQ-PDA-AUN	4T1 breast tumor	CD4+ and CD8+ T cells	Not analyzed	Primary tumor regression.	[151]
CD47			TAMs polarization from M2 to M1			
PDL1	Gold nanostar	Murine bladder cancer	CD4+ and CD8+ T cells	Not analyzed	Reduced primary tumor growth and distant metastasis. Long-term immunity in re-challenge experiments.	[145]
		MB49	B cells			
PDL1	Au@Pt nanoparticles	4T1 breast tumor	CD4+ and CD8+ T cells	IFNγ, IL-6, IL-12, TNFα	Regression of primary and distal tumors, inhibition of metastasis.	[152]
PDL1 and IDO	Reduced graphene oxide-based nanosheets	CT26 murine colon cancer	DCs, NK cells, CD45+ leukocytes, CD4+ and CD8+ T cells	IFNγ	Primary tumor regression and distant tumor regression by abscopal effect.	[153]
PDL1 and R837	Fe3O4-R837 spherical superparticles	4T1 breast tumor	DCs, NK cells, B cells, CD4+ and CD8+ T cells	IFNγ, IL-6, TNFα	Primary tumor regression and distant tumor regression by abscopal effect.	[154]
CTLA4	Single-walled nanotubes (SWNTs)	BF16-F10 murine melanoma	DCs, CD4+, CD8+, CD20+ T cells	IL-6, IL-12, IL-1β, TNFα	Reduced primary tumor growth and distant metastasis.	[146]
		4T1 breast tumor				
CTLA4	Prussian blue nanoparticles (PBNP)	Murine neuroblastoma cell Neuro2a	CD4+ and CD8+ T cells	Not analyzed	Lower tumor burden, synergistic effect on enhanced survival, development of immune memory in re-challenge experiments.	[155]
CTLA4 and R837	Indocyanine green and R837 co-encapsulated by poly (lactic-co-glycolic) acid (PLGA)	4T1 breast tumor	DCs, CD4+, CD8+ T cells, memory T cells	IL-6, IL-12, IL-1β TNFα, IFNγ	Primary tumor regression and distant tumor regression by abscopal effect; inhibition of metastasis.	[147]
		Colon cancer CT26				

**Table 3:** A list of selected pre-clinical studies using combinations of immune checkpoint inhibition and radiation therapy.

Checkpoint Inhibitor Target	Radiation Therapy Dose (Fractions)	Murine Tumor Model	Immune Effector Cells	Cytokines	Therapeutic/Immune response	Ref.
PD1	8 Gy (4 fractions)	Metastatic melanoma in the brain	CD8+ T cells	Not analyzed	Reduced tumor growth and systemic immunity by abscopal effect	[187]
PD1	24 Gy (3 fractions)	Non-small-cell lung carcinoma	Neutrophils, CD4+ and CD8+ T cells	IL-5, IFNγ, TNFα	Higher lung injury score, increased inflammatory response	[188]
PD1	16 Gy (2 fractions)	B16-F10 melanoma TS/A mammary adeno-carcinoma	DCs, monocytes, macrophages and CD8+ T cells	IFNβ upregulated in abscopal tumors	Reduced tumor growth and systemic immunity by abscopal effect	[189]
PDL1	12 Gy	Pancreatic cancer	CD4+ and CD8+ T cells, myeloid-derived suppressor cells, tumor-associated macrophages	Not analyzed	Reduced primary tumor growth and systemic immunity by abscopal effect	[190]
PDL1	10 Gy	Head and neck squamous cell carcinoma	CD4+ and CD8+ T cells	Not analyzed	Enhanced tumor control and improved survival	[191]
PDL1	10 Gy	Hepatocellular carcinoma	CD8+ T cells	Not analyzed	Significant suppression of tumor growth and improved survival	[192]
CTLA4 along with immature dendritic cells (iDCs)	10 Gy	Colon cancer CT26	IFNγ-secreting T cells, CD8+ CTLs	IFNγ	Suppression of tumor growth and improved survival of tumor-bearing mice	[193]
CTLA4	10 Gy	Orthotopic glioma	CD4+ and CD8+ T cells	Not analyzed	Improved survival of treated mice	[194]
PD1 + CTLA4	20 Gy (either single dose or in fractions)	4T1 mammary carcinoma	APCs, CD4+ and CD8+ cells	IFNγ	Primary tumor regression, abscopal effect in fractionated dose	[195]
PD1 + CTLA4	10 Gy	LM8 osteosarcoma	CD8+ T cells	Not analyzed	Reduced primary tumor growth and lung metastasis, systemic immunity by abscopal effect	[196]

**Table 4:** A list of ongoing clinical studies using a combination of immune checkpoint inhibition with radiation therapy [16,161].

Checkpoint Molecule Targeted for ICI	ICI Agent Used	Disease	Radiation Therapy Dose (Fractions)	Additional Drugs Used	Estimated Patient Accrual (*n*)	Timing of Radiotherapy	ClinicalTrials.gov for ICI Identifier *
PD1	Nivolumab	Glioblastoma	2 Gy × 30	Temozolomide	693	n/s	NCT02667587
PD1	Nivolumab	Glioblastoma	not specified	Temozolomide	550	n/s	NCT02617589
PD1	Pembrolizumab	HNSCC, locally advanced	2 Gy × 35	Cisplatin	780	ICI then RT (RT at cycle 2 of ICI)	NCT03040999
PD1	Nivolumab	HNSCC, locally advanced	n/s	Cisplatin, Cetuximab	1046	n/s	NCT03349710
PD1	Pembrolizumab	Breast cancer, triple negative	n/s	chemotherapy	1000	RT then ICI	NCT02954874
PD1	Nivolumab	NSCLC, Stage IV	4 Gy × 5	none	130	ICI then RT	NCT03044626
PD1	Pembrolizumab	Breast cancer, localized	8 Gy × 3 (alternate days)	± Flt3 ligand (CDX-301)	100	n/s	NCT03804944
PD1	Nivolumab	Pancreatic cancer (PDAC)	6.6 Gy × 5	± CCR2/CCR5 dual antagonist; ± GVAX	30	RT then ICI	NCT03767582
PD-L1	Durvalumab	Glioblastoma, recurrent	8 Gy × 3 once daily	none	62	RT then ICI (ICI starts on last day of RT)	NCT02866747
PD-L1	Durvalumab	Breast cancer, luminal B	SBRT 8 Gy × 2 fractions preoperatively	chemotherapy, ± anti-CD73 (oleclumab)	147	RT then ICI	NCT03875573
PD-L1	Avelumab	Hepatobiliary malignancy(advanced)	Hypofractionated in 5 fractions	DNA-PK inhibitor	92	RT then ICI	NCT04068194
PD-L1	Avelumab	Various advanced solid tumors	30 Gy in 10 fractions over 2 weeks	DNA-PK inhibitor	54	RT and ICI together (1st dose), then ICI continues	NCT03724890
CTLA4	Ipilimumab	Prostate cancer (metastatic)	n/s	none	988	RT then ICI	NCT00861614
PD1, and PD-L1	Nivolumab, and atezolizumab	RCC Stage IV, or UC Stage IV	3 Gy × 10	none	112	RT begins ±24 h of ICI start	NCT03115801
PD1, and CTLA4	Nivolumab, and Ipilimumab	NSCLC, Stage IV	n/s	none	270	ICI then RT	NCT03391869
PD-L1, and CTLA4	Durvalumab, and tremelimumab	NSCLC and colon cancer	High dose: 1 daily fraction × 3 days; Low dose: 2 fx daily on weeks 2, 6, 10, and 14	none	180	ICI then RT	NCT02888743
PD-L1, and CTLA4	Durvalumab, and tremelimumab	SCLC, relapsed	SBRT or hypofractionated RT over 3–5 days	none	20	RT then ICI	NCT02701400
PD-L1, and CTLA4	Durvalumab, and tremelimumab	SCLC, advanced stage	30 Gy in 10 fractions over 2 weeks	PARP inhibitor (olaparib)	54	RT then ICI	NCT03923270
PD-L1, and CTLA4	Durvalumab, and tremelimumab	Esophageal cancer, Stage III–IV	n/s	chemotherapy	75	ICI then RT	NCT02735239
Any ICI target	Any approved agent	Any metastatic cancer, with a lesion treatable with SBRT	SBRT 9.5 Gy × 3	none	146	ICI then RT	NCT02843165

* Verified on ClinicalTrials.gov (accessed on 24 April 2021). n/s, not specified.

## Abbreviations

Aminolevulinic acid-photodynamic therapy	ALA-PDT
Antigen-presenting cells	APC
Ataxia-telangiectasia mutated	ATM
Adenosine triphosphate	ATP
B7 protein family dendritic cell molecule	B7-DC
Bristol Mayers Squibb 202	BMS-202
Bursa of Fabricius cells	B cell
Calreticulin	CRT
Chemokine (C-X-C motif) ligand 2	CXCL2
Cluster of differentiation 8	CD8
Cytotoxic T lymphocyte	CTL
Cytotoxic T lymphocyte antigen 4	CTLA4
Damage-associated molecular patterns	DAMP
Damage-regulated autophagy modulator	DRAM
Dendritic cells	DC
Draining lymph nodes	DLN
External beam radiation therapy	EBRT
Forkhead box P3	FoxP3
Genetically engineered mouse model	GEMM
Glysated chitosan	GC
Gold nanorod	GNR
Gold nanostar	GNS
Granulocyte-macrophage colony-stimulating factor (GM-CSF)-transfected tumor cell vaccine	GVAX
Head and neck squamous cell carcinoma	HNSCC
Heat shock proteins	HSP
High mobility group box 1	HMGB1
Immune checkpoint blockade	ICB
Immune checkpoint inhibition	ICI
Immunogenic cell death	ICD
Indoleamine-pyrrole 2,3-dioxygenase	IDO
Interferon	IFN
Interferon gamma	IFNγ
Interleukin 1 beta	IL-1β
Interleukin 6	IL-6
Interleukin 12	IL-12
Interleukin 17	IL-17
Ionizing radiation	IR
Lymphocyte-activation gene 3	LAG3
Lipopolysaccharide	LPS
Monoclonal antibodies	mAbs
Macrophage inflammatory protein 2	MIP2
Magnetic Fe_3_O_4_ photothermal nanoparticle	MNP
Major histocompatibility complex I and II	MHC I and II
MHC class I polypeptide-related sequence A	MICA
Myeloid-derived suppressor cells	MDSC
Natural killer	NK
Natural killer group 2D	NKG2D
Near-infrared radiation	NIR
Non-small-cell lung carcinoma	NSCLC
Organic semiconducting pro-nano stimulant	OSPS
Pancreatic ductal adenocarcinoma	PDAC
Pattern recognition receptors	PRR
Photodynamic therapy	PDT
Photosensitizer	PS
Photothermal agents	PTA
Photothermal therapy	PTT
Poly(lactic-co-glycolic) acid-indocyanine green-R837	PLGA-ICG-R837
Programmed cell death protein 1	PD1/PDCD1
Programmed cell death protein 1 ligand 1	PDL1
Prussian blue nanoparticle	PBNP
Radiation therapy	RT
Reactive oxygen species	ROS
Regulatory T cells	Treg
Renal cell carcinoma	RCC
Stereotactic body radiation therapy	SBRT
Small-cell lung cancer	SCLC
Single-walled carbon nanotubes	SWCNT
Silica–gold nanoshell	AuNS
T cell receptor	TCR
T helper 17	Th17
T lymphocytes	T cell
Toll-like receptors	TLR
Transforming growth factor beta	TGFβ
Tumor-associated macrophages	TAM
Tumor microenvironment	TME
Tumor necrosis factor alpha	TNFα
Tumor neoantigens	TNA
Tumor-specific antigens	TSA
Urothelial carcinoma	UC
Vascular endothelial growth factor	VEGF
